# Effects of Vibrotherapy with Different Characteristics and Body Position on Post-Exercise Recovery after Anaerobic Exercise

**DOI:** 10.3390/jcm12144629

**Published:** 2023-07-12

**Authors:** Tomasz Pałka, Marcin Maciejczyk, Olga Czerwińska-Ledwig, Łukasz Tota, Marek Bawelski, Alejandro Leiva-Arcas, Rafał Stabrawa, Przemysław Bujas, Dawid Mucha, Andrzej Wiśniewski, Anna Piotrowska

**Affiliations:** 1 Department of Physiology and Biochemistry, Faculty of Physical Education and Sport, University of Physical Education in Krakow, 31-571 Krakow, Polandmarcin.maciejczyk@awf.krakow.pl (M.M.); marek.bawelski@awf.krakow.pl (M.B.); 2 Department of Chemistry and Biochemistry, Faculty of Physiotherapy, University of Physical Education in Krakow, 31-571 Krakow, Poland; 3 Faculty of Sport, San Antonio de Murcia Catholic University Los Jerónimos Campus, 30107 Guadalupe, Spain; 4 Institute of Physical Education, State Higher School of Vocational Education, 33-300 Nowy Sącz, Poland; 5 Department of Sports Theory and Anthropomotorics, University of Physical Education in Krakow, 31-571 Krakow, Poland; 6 Department of Medicine and Health Sciences, Andrzej Frycz Modrzewski Krakow University, 30-705 Kraków, Poland; 7 II Department of Internal Medicine and Cardiology, Stefan Żeromski Specialist Hospital, 31-913 Krakow, Poland

**Keywords:** tonic vibration reflex, vibrotherapy, post-exercise recovery

## Abstract

The aim of this project was to indicate the optimal parameters such as frequency, duration of a single vibrotherapy, and body position, which will be used as a form of recovery modality after physical exercise. Sixteen healthy male volunteers were involved in this study. The aerobic and anaerobic capacity of participants was assessed. Each of the subjects performed a set of intensive physical exercises and then underwent vibrotherapy treatment. In random order, each of the men tested the effectiveness of eight of the combinations of frequency, duration, and body position. The effect of the procedure accelerating recovery was assessed 24 h after physical exercise with the Wingate test. Changes in oxygen saturation and biochemical markers (interleukins: Il-1β, Il-6, and creatine kinase: CK), hemoglobin (Hb), and hematocrit (Hct) were assessed 1 h and 24 h after the physical effort. Lactate concentrations were measured 3, 15, 30, and 60 min after the end of the vibration. It was indicated that the optimal treatment should be based on lower ranges of frequency values (2–52 Hz). The procedure with raised feet is also more beneficial than the flat, supine position. To improve the overall work, and a number of biochemical markers (CK and Il-1β), a 45 min treatment will be more efficient, because significantly lower CK activity was indicated for the 45 min treatment. For this duration, higher values of Il-1β were indicated in the measurement carried out for samples collected 60 min after the treatment and lower in the measurement carried out 24 h after the treatment.

## 1. Introduction

Physical effort is a strong stressor modifying the functional state of the human body [1]. It also has an effect on the emotional state [2]. Therefore, it is substantially important to return to the optimal psychophysical state post-stress as soon as possible, which is especially important in the restrictive training of professional athletes. Currently, a number of methods are used for this purpose, and most scientific data indicate the usefulness of massage [3] and cryotherapy [4]. Another researched activity in this field is vibrotherapy [5,6,7,8,9,10,11,12].

Originally, vibration stimulus was used for healing and therapeutic purposes in order to achieve an analgesic effect. The studies performed in the following years showed that vibration can also have other beneficial effects, e.g., metabolic processes regulation, reduction of fibrinogen levels, normalization of the vascular system, and activation of muscle spindles (for review, see [13,14,15,16,17]). Subsequent projects have also shown the safety of this physical stimulus, and the list of contraindications for the use of treatments based on vibration is relatively short [18]. Contraindications to treatments using a vibration stimulus were divided into those that occurred in the patient in the past (metastatic malignant tumors, severe cardiovascular diseases, thrombosis, severe surgical interventions involving the musculoskeletal system, acute back pain, advanced diabetes with episodes of hypoglycemia), currently present (pregnancy and lactation, pacemaker, acute inflammation, recovery period after endoprosthesis of the hip or knee joint), and those that appeared during the vibration treatment (headache, dizziness, nausea) [18].

On the other hand, vibration is an environmental risk factor and is an important issue of occupational medicine. It was shown that exposure to vehicular vibration causes fatigue and reduced attention in driving tasks [19]. Two stressors related to the work environment are often combined: noise and vibration. It was indicated that single and combined exposure to these stressors had mainly negative effects on auditory attention, while the effect on visual attention was ambiguous [20]. The distinction between the stressful and therapeutic effects of a vibrational stimulus depends on the vibrational parameters. This mechanical stimulus is transmitted in the body in the form of waves, characterized by amplitude, frequency, duration, and direction. ISO standards for vibration as an environmental factor permitted in the workplace for health, comfort, and perception indicate frequencies in the range from 0.5 Hz to 80 Hz [21]. The most popular and, so far, the best-studied form of using vibrations is whole-body vibration (WBV) [22]. It is a method used both in rehabilitation and sports medicine, as well as in sports training.

An alternative to WBV is to use locally applied vibration [7]. Devices generating a vibrating stimulus applied to the surface of the body or adapted to place on the surface of the body to transmit vibrations are used for this purpose [18].

Regardless of the form of vibration stimulus applied, several mechanisms of vibration action on the human body are indicated: tonic vibration reflex [17,23]; influence on blood flow in the vascular bed through rheological action and increased release of vasodilating factors; influence on the conduction of nerve signals in the posterior horns of the spinal cord; and other [18].

The available literature indicates that WBV is effective in reducing delayed onset muscle soreness (DOMS) after intense exercise with a predominance of eccentric contractions [5,6,7,8,9,10,11,12]. However, available studies differ in terms of used protocols, methods, and obtained results. Aminian-Far et al. [9] showed that the use of WBV after a series of eccentric exercises was associated with a reduction in DOMS symptoms, plasma creatine kinase (CK) levels, pressure point threshold, and muscle soreness. In the study of Lau et al. [5], the vibration did not eliminate swelling and did not contribute to the regeneration of muscle strength and any change in CK activity in the serum. Low-frequency (5 Hz) WBV reduced levels of soreness and muscle tension or cramping after intense training [8,11]. Vibration (frequency 35 Hz; amplitude 2 mm) has been shown to reduce muscle pain, tenderness, and stiffness [11]. In 2021 [10], a study in which elite hockey players participated was conducted. The subjects underwent an eccentric exercise protocol followed by WBV or stretching. After WBV, lower pain ratings and lower quadriceps tension were indicated. It has also been shown that WBV can be used as a warm-up to reduce the risk of DOMS before exercise [6,24].

The aim of our previous study [7], in which 12 untrained men took part, was to demonstrate the effect of local vibration therapy applied after exercise on a range of selected biomarkers used to quantify muscle fiber damage. After 180 min of exercise with an intensity of 50 ± 2% VO2peak, the subjects underwent a 60 min treatment (in a semi-recumbent position) on a vibrating mattress or a placebo. Immediately after the hour-long recovery procedure, myoglobin (Mb) concentrations and CK and LDH (lactate dehydrogenase) activities were compared. Significantly lower values of the tested markers were observed in the vibration group. Differences also occurred 24 h after the end of the exercise test. In a similar protocol, the effect of local vibrotherapy on post-exercise plasma metalloproteinase 2 (MMP-2) concentrations was investigated. Again, it was shown that the use of vibrotherapy increases the rate of post-exercise recovery of the body [25].

The papers mentioned above confirm that vibration is an important stimulus that affects the speed of post-exercise recovery. However, depending on the study protocol and the characteristics of the stimuli used, the results vary. This problem is a frequently raised topic in vibration studies. The latest guidelines show how data from studies on the use of vibrotherapy in humans and animals should be reported [26]. Compliance with them will be an important element in facilitating meta-analyses of data obtained in individual research studies.

The aim of this study was to indicate the optimal parameters of vibrotherapy such as frequency, duration, and treatment position, which can be used as an effective recovery modality after prolonged physical exercise. We hypothesized that the effectiveness of WBV in recovery may depend both on its characteristics (frequency and duration) and on the position of the body during the treatment.

## 2. Materials and Methods

### 2.1. Study Design

This research project was approved by the Bioethics Committee at the Regional Medical Chamber in Krakow47/KBL/OIL/2022 on 11 April 2022. In accordance with the requirements of the Declaration of Helsinki, participants were informed about the purpose of the research, the methods used, possible side effects, and the possibility of resigning from participation in the research at any stage without giving a reason. Participants gave written consent to participate in the study and all procedures to be performed. During the tests, the participants of the project were supervised by properly trained medical staff.

**Figure 1:**
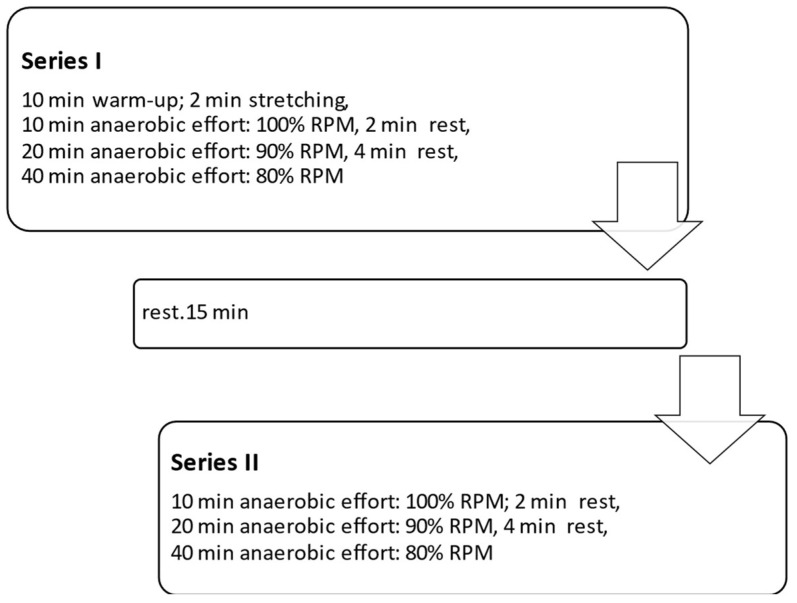
Scheme of anaerobic efforts. %RPM—percentage of maximum pedaling frequency.

Devices generating vibrations in the lower (I: 2–52 Hz) and higher frequency ranges (II: 82–100 Hz) (Vitberg, Nowy Sącz, Poland, registration number HD 1497948-1) were used for the tests. The basic methodology of the procedure was similar to that described in our previous paper [7]. The main differences were the use of devices with different vibration parameters, different treatment positions, and treatment times. The treatment durations were either 10 min (T1) or 45 min (T2), as well as two body positions during the vibrotherapy: lying down with lower limbs raised by 20° (body position A) and lying down (body position B). The participants performed two series of anaerobic efforts with a rest break, according to the scheme shown in Figure 1.

**Figure 2:**
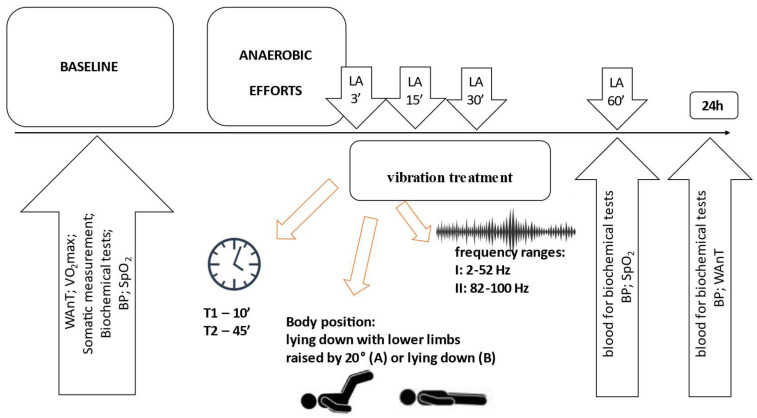
General scheme of the research. LA—lactate assessment; WAnT—Wingate Anaerobic Test; VO_2_max—maximal oxygen uptake; BP—blood pressure; SpO_2_—oxygen saturation; T—time.

After two series, the subjects underwent a vibration treatment in 1 of the 8 tested combinations (as per Figure 2): I-T1-A, I-T2-A, I-T1-B, I-T2-B, and II-T1-A, II-T2-A, II-T1-B, and II-T2-B. Each participant was subjected to all combinations (in random order). Testing of individual combinations after anaerobic exercise was carried out in 10-day intervals, necessary for the expiry of possible effects of physical effort and vibration.

Twenty-four hours after completing each of the eight-test series, the men performed a 30 s Wingate test [27] assessing anaerobic capacity (each participant completed the Wingate test 8 times).

### 2.2. Participants

From the group of volunteers, 16 healthy men were selected, who were characterized by a similar level of physical fitness described by the ACSM (The American College of Sports Medicine) as fair with VO_2_max 44.9–50.1 mL·kg^−1^·min^−1^ [27]. The following inclusion criteria were applied: male sex, age between 20 and 30 years, and body composition parameters within the normal ranges for the Polish population [28,29]. The exclusion criteria included any contraindications to physical activity and contraindications to the use of vibration [18]. During the study period, they did not consume any stimulants and did not use vitamins or dietary supplements and did not change their eating habits or recreational physical activity. All participants were students of physical education and performed physical activities related to the study program. The acceptable additional physical effort included a maximum of 2 h of moderate-intensity activities per week. The description of the study group is presented in Table 1.

All the exercise tests involving study participants were carried out in an air-conditioned laboratory (ambient temperature 21 °C and relative humidity of about 40%; Harvia thermohygrometer (Finland) and Ellab electrothermometer (Denmark) with an accuracy of 0.5 °C and 3%, respectively) in the morning, not earlier than 2 h after a light meal. The research was divided into two parts: preliminary (stages I and II) and main (stage III).

### 2.3. Somatic Measurements and Exercise Tests

In the first stage, a medical screening was carried out, and blood pressure (BP) and heart rate (HR) were measured. Arterial blood pressure (BP) at the level of the brachial artery was measured in the sitting position with an accuracy of 5 mmHg (0.67 kPa). Oxygen saturation (SpO_2_) was measured with a Welch Allyn^®^ Spot Vital Signs^®^ 4400 (accuracy ± 1%) with Suretemp and a Pulse Oximeter (Nonin Medical Inc., Plymouth, MA, USA) (accuracy ± 2%).

Body height (BH; Seca stadiometer, Germany) was measured with an accuracy of 0.5 cm) and body mass (BM; Sartorius type F 1505—DZA; Germany) with an accuracy of 100 g, BMI was calculated, and body composition was estimated based on bioimpedance measurements. The analysis of body fat percentage (PBF), fat mass (FM), and lean body mass (LBM) was performed using the body composition analyzer JAWON MEDICAL IOI-353 (Republic of Korea) with EC0197 certificate.

In stage II, the men underwent exercise tests assessing anaerobic and aerobic capacity. After seven days, the subjects proceeded to basic research (stage III).

The Wingate test [27] was used to assess anaerobic capacity parameters. The main effort was preceded by a 5 min warm-up on a cycloergometer with individually selected intensity at a frequency of 60 rpm with 3 to 5 s maximal accelerations in the 2nd, 4th, and 5th minute. Two minutes after its completion, participants performed a thirty-second supramaximal physical effort. The task of the tested person was to achieve the maximum rhythm of pedaling in the shortest possible time and maintain it as long as possible. The external resistance was 7.5% of the body mass of the participant. During the test, mean power (MP), total work (TW), peak power (PP), power decrease indicator (IDP), time to obtain peak power (toPP), and time to maintain peak power (tmPP) were analyzed (MCE, Staniak, Poland). The physical warm-up preceding the anaerobic test was performed on a Monark 827E bicycle ergometer (Sweden), and the main part of the test was on a Monark 875 E ergometer (Sweden).

After a minimum of two hours from the end of the Wingate test, the participants performed the incremental test; the test assessed aerobic capacity. A maximal intensity test to volitional exhaustion started with a 2 min warm-up on a cycloergometer with a pedaling frequency (RPM) of 60 revolutions per minute and an intensity of 110 W. Then, every 2 min, the power was increased by 20 W. The effort lasted until the subjective feeling of inability to maintain the desired pedaling rhythm. During the graded test, the following respiratory exchange indices were recorded in 30 s intervals: oxygen consumption (VO2), carbon dioxide production (VCO2), respiratory exchange ratio (RER), respiratory rate (FR), tidal volume (TV), and pulmonary ventilation (VE) using an ergospirometer (Cortex Metalyzer, Germany). The maximal power (MWL) was also determined. Heart rate (HR) was continuously recorded by telemetry (Polar, Finland). The graded test was performed on a cycle ergometer type ER 900 D—72475 BIT2 by Jeager (Germany).

Results of somatic measurements and aerobic and anaerobic tests at baseline are presented in Table 1.

### 2.4. Blood Sampling and Biochemical and Hematological Analyses

Blood for biochemical and hematological parameters evaluation was collected in accordance with the applicable standards by a laboratory diagnostician from the antecubital crease at the elbow. Blood was collected before (baseline), and 1 and 24 h after exercise.

For hematological parameter determinations, 5 mL of blood was collected into a tube with EDTA (ethylenediaminetetraacetic acid, disodium salt). In whole blood collected, the hematocrit (Hct) and hemoglobin (Hb) were determined. The Hct used to calculate changes in plasma volume was determined by the micromethod using a Unipan centrifuge type MPW—212H (Poland). The concentration of Hb (g/dL^−1^) in venous blood was determined with the use of the Drabkin method. For biochemical determinations, the blood was collected in 6 mL tubes containing a clot-activating agent. After collection, the blood was centrifuged in a laboratory centrifuge (MPW 351R Med. Instruments Polska). The serum was separated from the clot and frozen at −70 °C (Artico ULF 390 PRC, Denmark) until further analysis (no longer than 3 months). Concentrations of interleukin 1β (IL-1β) and interleukin 6 (IL-6) creatine kinase CK activity and myoglobin (Mb) concentration were determined using the immunoenzymatic method using the E-Liza Mat 3000 microplate reader (DRG instruments GmbH, Germany) with use of commercially available kits (DRG Instruments GmbH, Germany).

Changes in plasma volume (%ΔPV) were calculated using the Dill and Costill [30] formula with Harisson’s modification [31]:

%ΔPV = 100 {(Hb1/Hb2) · [100 − (HCT2 · 0.874)]/[100 − (HCT1 · 0.874)] − 1}(1)

where Hb1 and HCT1 are the baseline levels of hemoglobin and hematocrit, Hb2, and HCT2 are the values obtained after physical effort.

Post-exercise biochemical concentrations were corrected for changes in plasma volume. The Kraemer and Brown [32] formula was used to calculate the corrected values.

Vc = (%ΔPV · 0.01 · V2) + V2(2)

where Vc—corrected value and V2—value obtained after physical effort.

In addition, before and after 3, 15, 30, and 60 min from the end of the vibro-treatment, the concentration of lactate (LA) in arterialized blood samples was determined. Ten microliters of blood was collected from the fingertip of the fourth finger into a heparinized capillary tube. The concentration of LA was determined by the enzymatic method using the Miniphotometer plus DR Lange, type LP-20 by Dr. Lange (Germany).

### 2.5. Statistical Analysis

The results for each combination are presented using basic descriptive statistics. Statistical analysis was performed using the Statistica 13 software (SoftStat, Poland). A two-way MANOVA was used to examine the interaction between the repeated measure factors and between-group factors (frequency × treatment duration × item). If significant interactions were found, probabilities were calculated for post-hoc tests using the LSD test. In order to determine the effect size, partial eta squared (η2P) was calculated, whose values > 0.01, 0.06, and 0.14 correspond to a small, medium, and large extent of the effect size, respectively. The results were considered significant for *p* < 0.05.

## 3. Results

### 3.1. Physiological Parameters

Regardless of the frequency, duration of the procedure, or body position, significant differences in systolic blood pressure were found (F = 4.983; *p* = 0.008; ηp2 = 0.041). The post-hoc test showed the difference of baseline vs. measurements at 60 min after exercise, when blood pressure values were significantly lower. The importance of the frequency, the duration of the vibration treatment, or the body position was not indicated.

Similar results were obtained for diastolic blood pressure (F = 13.909; *p* > 0.001; ηp2 = 0.105). The lowest values were found 24 h after exercise. No differences were indicated for treatments in different body positions or the duration of a single procedure.

Changes over time were indicated for heart rate (HR) (F = 54.494; *p* < 0.001, ηp2 = 0.316). Significantly higher values were recorded 60 min after exercise. The importance of vibration characteristics, treatment characteristics, and significant interactions were not indicated.

For the level of blood oxygen saturation (SpO_2_), the importance of the duration of the procedure was indicated (F = 3.865; *p* = 0.022; ηp2 = 0.032). For treatments lasting 45 min, a decrease in SpO_2_ was recorded after 60 min and 24 h after exercise. However, in the case of treatments lasting 10 min, the opposite situation was observed; SpO_2_ values increased both 60 min and 24 h after exercise. The basic characteristics of the variables discussed here are presented in Table 2.

### 3.2. Wingate Test

Statistically significant differences in total work (TW) were indicated (F = 1046.106; *p* < 0.002; ηp2 = 0.897). It was observed that the baseline values differ from the values determined 24 h after the vibration treatment; after 24 h, lower values of this variable were indicated (by 72.7 kJ). Statistically significant interactions were indicated for measurement–frequency (F = 6.114; ηp2 = 0.048; *p* = 0.015), where the interaction with the frequency I after 24 h brought higher results than with the interaction with the frequency II; measurement–time (F = 4.288; *p* = 0.041; ηp2 = 0.034), where treatment duration of 45 min gave higher results compared to treatments lasting 10 min; and measurement–position (F = 6.683; *p* = 0.011; ηp2 = 0.053), where in position A (lying down, legs up 20°) after 24 h, the results were 11.6 kJ, higher compared to position B (lying down).

No statistically significant differences were found in the peak power (PP) and relative peak power (RPP) measurements.

The time to obtain maximum power significantly differed, with differences in baseline vs. 24 h from the vibrotherapy session (regardless of the frequency, duration of treatment, or body position) (F = 40.225; *p* < 0.001; ηp2 = 0.251). Twenty-four hours after the procedure, a shorter time to reach maximum power was noted. However, no significant interactions between the measurements and the frequency, duration of treatments, and position were indicated.

Similar results were found for the time of maintaining maximum power; the subjects obtained significantly lower results 24 h after the vibration treatment compared to the baseline (F = 25.572; *p* < 0.001; ηp2 = 0.176). Again, the role of the characteristics of the procedure was not indicated here.

In the case of the power decrease index, significantly higher values of this variable were recorded 24 h after the procedure compared to the initial measurement (F = 113.471; *p* < 0.001, ηp2 = 0.486). Wingate test indices measured 24 h after exercise are shown in Table 3.

### 3.3. Biochemical Parameters

Statistically significant differences in Mb concentrations over time were indicated (F = 159.743; *p* < 0.001; ηp2 = 0.571). Higher scores were shown 60 min after treatment compared to baseline and to results obtained 24 h after treatment. There were no differences between the measurements 60 min and 24 h after the procedure. In addition, a statistically significant measurement–frequency interaction was noted (F = 4.305; *p* = 0.022; ηp2 = 0.035). Frequency I showed lower myoglobin levels 60 min after treatment compared to frequency II. There were no significant interactions between measurement and position and measurement and treatment duration.

Statistically significant differences in CK concentration between the measurements were shown (F = 13.622; *p* < 0.001; ηp2 = 0.102). The post-hoc test indicated a statistically significantly lower baseline vs. 60 min and 24 h after treatment. There were no differences between the measurements 60 min and 24 h after the procedure. In addition, a statistically significant interaction between measurement and procedure duration was noted (F = 7.712; *p* = 0.001; ηp2 = 0.060). The 45 min treatment showed lower values when measured 24 h after the treatment compared to the 10 min treatment. There were no statistically significant differences between the other measurements. There were also no significant measurement–frequency and measurement–position interactions.

IL-1β concentrations significantly differed over time (F= 87,519; *p* < 0.001; ηp2 = 0.422). Higher serum concentrations were indicated 60 min after the procedure compared to the measurements taken immediately before the procedure and those obtained 24 h after the procedure. In addition, 24 h after the procedure, a significantly lower value was obtained compared to results obtained immediately after the procedure. There was also a statistically significant interaction between measurement and procedure duration (F = 4.745; *p* = 0.018; ηp2 = 0.038). For the 45 min treatment, higher values were indicated in the measurement carried out for samples collected 60 min after the treatment and lower in the measurement carried out 24 h after the treatment. There were no statistically significant differences between the other measurements. There were no significant measurement–frequency and measurement–position interactions.

In the case of IL-6, no statistically significant differences between the measurements and the studied measurement–frequency interactions were indicated, as well as for measurement–position and measurement–treatment duration (*p* > 0.05). The variables discussed here are presented in Table 4.

### 3.4. Lactate Concentration in Arterialised Blood

**Figure 3:**
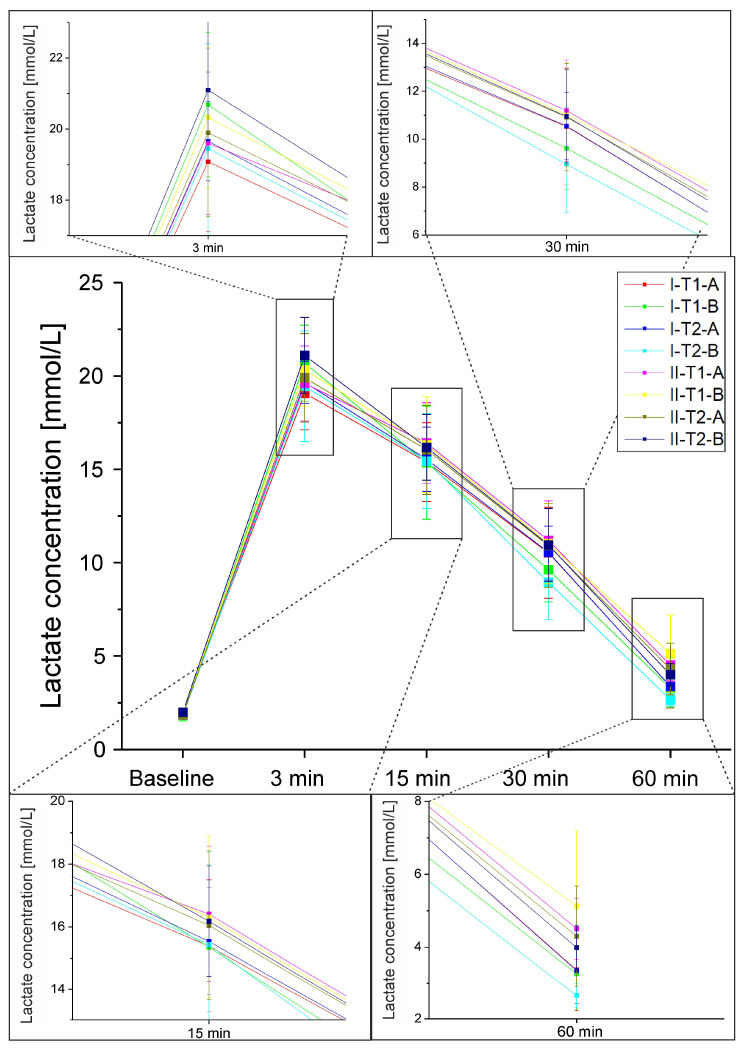
Lactate concentration in the arterialized blood for individual sets of frequency of vibrations/body position/duration of the vibrotherapy procedure. Frequency of vibrations: I: 2–52 Hz, II: 82–100 Hz; vibrotherapy duration: T1: 10 min, T2: 45 min; body position: A: lying down with lower limbs raised by 20°, B: lying down.

The results of lactate concentration in the blood obtained from the fingertip for individual sets of frequency of vibrations/body position/duration of the procedure are presented in Figure 3. Statistically significant differences in lactate concentration between subsequent measurements were indicated (F = 3783.524; *p* < 0.001, ηp2 = 0.971). Significant differences between all performed measurements were indicated (*p* < 0.001). The lowest concentrations were indicated in the measurement carried out before exercise, followed by the results from the measurement carried out 60 min after the vibration treatment, 30 min after, and then, 15 min after. The highest values were indicated immediately after the procedure. Statistically significant interactions were indicated: measurement–frequency (F = 3.786; *p* = 0.005; ηp2 = 0.033), where, with interaction with lower frequencies, a smaller increase in lactate concentration was observed; measurement–position (F = 4.922; *p* = 0.003; ηp2 = 0.042), with higher lactate concentrations immediately after the procedure in the case of position B (lying down); no differences were noted for the other measurements. There were also no significant differences between the duration of the procedure and lactate concentration.

### 3.5. Hematological Blood Parameters and Plasma Volume Changes

Changes in Hb concentration and HCT values over time for various vibration treatments are shown in Table 4. Differences in Hb concentration over time were indicated (F = 4.336; *p* = 0.014; ηp2 = 0.035). Significantly higher values of hemoglobin concentrations were recorded in the measurement before the exercise compared to the measurements 60 min and 24 h after the exercise. Interactions between the measurements and the applied frequency, duration of treatments, and position were not indicated.

Statistically significant HCT differences between the measurements were indicated (F = 23.484; *p* < 0.001; ηp2 = 0.164). Higher HCT was observed before exercise compared to measurements 60 min and 24 h after exercise. A statistically significant interaction between measurement and position was also noted, where higher values were recorded 60 min after the procedure in the case of position B (lying down); no differences were noted in the case of other measurements. The importance of the treatment position was also not indicated.

There were no statistically significant differences in the change in plasma volume, taking into account the frequency of the procedure, the position, and the duration of the procedure.

## 4. Discussion

The present study provides data informing which set of vibration parameters and treatment options would be optimal for accelerating post-exercise recovery. The performed assessment of BP and HR did not indicate the optimal frequency or duration of position during the vibrotherapy in the study. The effect on blood pressure would require longer exposure to the stimulus (e.g., a series of vibro-treatments); therefore, it is not surprising that there are no interchangeable results using this endpoint. When assessing the level of blood oxygen saturation, it is suggested to use treatments lasting 10 min (differences of average effect size). No significant differences could be identified for the remaining features examined.

The Wingate test indicated that the use of treatments with frequency I (lower frequencies), lasting 45 min, in position A, results in higher values of total work. The maximal power, the time to reach maximal power, the time to maintain maximal power, and the power decrease rate for each set of treatment characteristics remained unchanged.

The results of biochemical analyses turned out to be a sensitive tool for differentiating the effects of using vibrotherapy as a form of accelerating post-exercise recovery. For frequency I, lower myoglobin concentrations 60 min after treatment were shown compared to frequency II (medium effect strength). As with blood oxygen saturation, it was indicated that the use of treatments with lower frequency parameters (I) gives better results.

The 45 min treatment led to significantly lower CK levels measured 24 h after treatment compared to the 10 min treatment (mean effect strength).

IL-1β concentrations varied significantly over time, which is a characteristic feature of muscle-damaging forms of physical effort [33,34,35]. Using the concentration of IL-1β as a marker of the effectiveness of vibrotherapy, our results suggest using treatments lasting 45 min. Prolonged action of the vibration stimulus through the effect on blood vessels (which affects the release of nitric oxide (NO) [36,37]), seems to accelerate the redistribution of cytokines and other markers of muscle fiber damage to the liver, where their metabolism will be possible. Earlier publications showed that this mechanism should be indicated as one of the most important effects of vibrotherapy, which will allow for the acceleration of post-workout recovery [7,25,38].

Another evaluated cytokine, IL-6, changed its post-workout concentration in a similar way as indicated in similar studies [39]. Skeletal muscles produce and release significant amounts of this interleukin, especially after prolonged physical exertion. Therefore, it is considered a myokine. This cytokine is also associated with muscle atrophy [40]. Previous studies have shown that vibrotherapy has a beneficial effect on the concentration profile of Il-6 [41].

The applied treatments influenced the post-exercise increases in arterialized blood lactate concentration in various ways. The obtained results suggest that vibrotherapy with frequency I in position A gives better results. It is not possible to indicate the optimal duration of the treatment because both the 10 min and 45 min treatments gave similar results for this variable.

The treatment position with raised feet seems to be a more effective position. Lower lactate values were indicated for this body position during the vibration treatment. This may be due to facilitated lymphatic drainage and improved redistribution of metabolites in the body [12]. The differences in the observed lactate concentrations may have been influenced by the level of blood flow through the vascular bed of skeletal muscles. The flow rate can be affected by factors such as the impact of physical work performed during the exercise test, the vibration stimulus used during rest after exercise, and the position of the body during the vibrotherapy treatment. With the cessation of exercise, blood flow in the exercised limbs rapidly decreases. This may result in locally increased lactate accumulation. The complete return of blood flow to the resting level is a long-term process and lasts more than 20–30 min [42]. There are three main parameters related to post-exercise blood flow intensity: peak blood flow, the time course of blood flow return to resting level, and post-exercise hyperemic blood volume. With this value, we measured the volume of blood that exceeds the resting flow flowing through the limb during the post-exercise period. After exercises of similar type, intensity, and duration, the congested blood volume in the exercised limb closely correlates with the heart rate at the end of the exercise. After a light workload, more congestion occurs in athletes than in non-training people. Significantly increased congestion in trained people compared to non-training ones was observed for various forms of exercise. This post-exercise increased congestion may occur due to less vasoconstriction in athletes compared to non-training men. Interestingly, the vibratory stimulus also affects the levels of a number of vasodilating factors, which may affect skeletal muscle congestion [18]. The differences observed in the previously cited study observed in training and non-training people may be conditioned by reduced activation of the sympathetic nervous system and vasoconstriction generated by the sympathetic impulse. This same mechanism is a known mechanism of vibrotherapy action [18]. The combination of the described effects related to the vascular bed with body position (lying position with raised limbs) and the action of gravity may have a significant role in accelerating the redistribution of lactates and myokines and their enzymatic conversion in the liver.

This study, however, did not indicate statistically significant differences in the change in plasma volume, taking into account vibration frequency, position, and duration of the procedure.

Summing up these results, it should be pointed out again that vibration is an effective method of accelerating post-exercise recovery; however, proper treatment parameters should be maintained. Increased proprioceptive function in the neuromuscular system, muscle strength, and hormonal and anti-inflammatory responses lead to pain reduction. Lymphatic drainage mediated by vibrations is also of great importance in eliminating DOMS. This will potentially make the effect of vibration similar to that of a massage [43].

The body position and the characteristics of the applied vibrations are important not only in the use of vibrotherapy in post-workout recovery, but also in rehabilitation. Vibrations of musculotendinous regions of a limb provoke illusionary sensations of movement. These ‘kinesthetic illusions’ gives beneficial results when used as a rehabilitation technique. Schofield et al. [44] conducted a study to characterize the effects of the vibration amplitude (0.1 to 0.5 mm), frequency (70 to 110 Hz), and limb position on the kinesthetic illusion (subjectively evaluated). It was shown that amplitude has the most profound impact on the kinesthetic illusion in the experimental ranges tested. The significant role of body position was also indicated. Frequency demonstrated no statistical effect. For vibrations with larger amplitudes, the effect depends on the shifts of individual tissues in relation to each other felt by the touch, pressure, and vibration receptors. In addition, because the change in amplitude is more subjectively felt, the importance of the frequency of the applied vibratory stimulus will not be so easy to assess in this way. This effect is confirmed by a study by Simsek et al. [45], where it was shown that both amplitude and frequency will be important modifiers indicating how vibration can modify training conditions by affecting the electromyography of muscle work. It is the effect on muscle fibers combined with the effect on the vascular bed that is, in our opinion, the basic mechanism of vibrotherapy.

### Study Strengths and Limitations

The main strength of the present study is the inclusion of many parameters of the vibration treatment. All combinations of treatment parameters were evaluated in the same subjects after a washout period, so we were able to obtain valuable results despite the small number of participants.

The basic limitation of our study is the small size of the study group and the lack of a control group. Due to the cost constraints of the study and the limited number of participants, the studies were conducted only on men to avoid sex variability. Therefore, another limitation of this study is that the results of this study apply only to men with this same level of physical fitness. All participants were asked not to change the form and level of recreational physical activity. Despite the inclusion criterion, which was a maximum of 2 h of moderate-intensity activity per week, it should be noted that leaving recreational physical activity uncontrolled is another limitation of this study.

## 5. Conclusions

An important effect of the vibration stimulus is the acceleration of the redistribution and elimination of pro-inflammatory molecules, the inhibition of local inflammation, and, consequently, the acceleration of the body’s readiness to make another effort. We indicate that the optimal treatment that can accelerate this process should be based on lower ranges of frequency values (2–52 Hz rather than 82–100 Hz). The procedure performed with raised feet is also more beneficial, which seems to increase drainage and elimination of inflammatory components. Assessing the level of oxygen saturation, a 10 min treatment will be more beneficial. However, to improve the overall work, and a number of biochemical markers, a 45 min treatment will be a better choice. Although, due to significant differences in the impact of the proposed vibration protocols on blood oxygen saturation, further research seems necessary.

## Figures and Tables

**Table 1:** Age, body composition, and aerobic and anaerobic performance at baseline in participants.

Variable	Min	Max	$\bar{x}$	SD
**Basic anthropometric characteristics**				
Age (years)	20.90	26.50	22.17	1.40
Body height; BH (cm)	166.20	186.10	175.70	5.64
Body mass; BM (kg)	64.00	91.30	74.74	7.60
Lean body mass. LBM (kg)	51.50	79.50	61.94	7.24
Soft lean mass. SLM (kg)	47.80	74.10	57.57	6.80
Total body water; TBW	37.10	57.20	44.59	5.20
BMI (kg/m^2^)	22.00	29.60	24.21	2.24
Percentage of body fat; PBF (%)	11.20	21.90	17.20	2.89
Fat mass; FM (kg)	8.10	18.00	12.79	2.25
Visceral fat area; VFA (cm^2^)	34.00	71.00	55.56	9.67
Proteins	10.70	16.90	12.98	1.60
Minerals	3.70	5.40	4.38	0.46
**Physiological indicators determined in the aerobic and anaerobic tests**				
Respiratory rate; BF (1 min^−1^)	36.70	80.30	54.86	11.29
Max. heart rate; HRmax (beats·min^−1^)	158.00	203.00	185.69	11.79
Max. pulmonary ventilation; VEmax (L·min^−1^)	77.10	205.50	133.78	32.98
Respiratory exchange ratio; RER	1.00	1.55	1.28	0.15
Tidal volume; TV (L)	2.17	3.42	2.62	0.32
Max. oxygen consumption; VO_2_max (L·min^−1^)	2.72	4.46	3.47	0.47
Max. oxygen consumption; VO_2_max (mL·kg^−1^·min^−1^)	37.60	53.60	46.37	3.97
Maximal power; MWL (W)	260.00	390.00	316.25	36.81
Load capacity (kg)	4.80	6.85	5.60	0.57
Mean power; MP (W)	553.00	899.00	681.00	96.18
Mean power; MP (W·kg^−1^)	7.60	10.50	9.09	0.66
Total work; TW (kJ)	16.59	26.97	20.43	2.88
Total work; TW (J·kg^−1^)	229.00	314.00	273.56	19.46
Peak power; PP (W)	687.00	1159.00	871.75	136.62
Relative peak power; RPP (W·kg^−1^)	9.87	13.75	11.63	0.96
Power decrease indicator; IDP (W·kg·s^−1^)	0.15	0.33	0.26	0.05
Time to obtain peak power; toPP (s)	3.00	5.72	4.40	0.76
Time to maintain peak power; tmPP (s)	2.39	8.13	3.75	1.42

**Table 2:** Values of systolic (SBP) and diastolic (DBP) blood pressure, heart rate (HR), and blood oxygen saturation (SpO_2_) at rest in participants (n = 16) subjected to vibrotherapy obtained before (0) and 60 min and 24 h after intensive exercise effort. Variables are shown as mean values ± standard deviation (SD).

		I-T1-A	I-T1-B	I-T2-A	I-T2-B	II-T1-A	II-T1-B	II-T2-A	II-T2-B
SBP (mmHg)	0	116.00 ± 9.08	119.56 ± 9.27	120.31 ± 7.27	117.29 ± 9.92	117.82 ± 10.48	116.65 ± 9.11	117.47 ± 6.22	117.59 ± 8.85
	60 min	118.35 ± 9.93	119.13 ± 9.09	123.63 ± 5.26	116.76 ± 11.09	119.76 ± 10.63	120.47 ± 6.76	119.82 ± 6.54	123.06 ± 8.50
	24 h	118.41 ± 11.61	116.94 ± 8.87	117.25 ± 9.01	118.47 ± 7.67	115.71 ± 8.62	117.53 ± 7.87	115.41 ± 6.37	116.65 ± 7.71
DBP (mmHG)	0	71.88 ± 6.18	71.19 ± 4.87	73.25 ± 5.43	72.82 ± 4.88	71.24 ± 7.32	71.35 ± 5.66	70.53 ± 6.52	72.06 ± 6.23
	60 min	72.88 ± 5.60	72.56 ± 4.75	74.88 ± 5.03	72.88 ± 5.70	72.24 ± 4.98	73.29 ± 4.40	73.35 ± 4.97	73.88 ± 6.13
	24 h	71.88 ± 7.01	69.63 ± 6.12	69.25 ± 7.26	71.12 ± 4.64	69.94 ± 6.15	71.12 ± 4.95	69.47 ± 4.96	71.47 ± 6.51
HR (bpm)	0	74.35 ± 9.62	76.31 ± 6.80	75.69 ± 6.94	77.76 ± 7.82	79.41 ± 8.09	73.76 ± 7.22	75.47 ± 9.46	78.24 ± 8.00
	60 min	82.47 ± 4.98	85.00 ± 4.73	83.81 ± 5.34	82.71 ± 5.72	81.53 ± 4.71	82.59 ± 3.50	80.94 ± 6.16	80.29 ± 10.09
	24 h	76.82 ± 9.15	75.69 ± 8.93	73.75 ± 9.77	73.18 ± 7.95	76.00 ± 7.94	74.88 ± 9.08	73.06 ± 6.84	78.12 ± 6.27
SpO (%)	0	98.35 ± 0.61	98.31 ± 0.70	96.75 ± 2.84	97.41 ± 2.18	97.65 ± 2.42	98.00 ± 1.06	98.47 ± 0.80	98.24 ± 0.75
	60 min	98.29 ± 0.69	97.56 ± 1.63	97.81 ± 1.52	97.76 ± 1.09	98.18 ± 1.38	97.94 ± 1.09	97.94 ± 1.43	97.88 ± 1.73
	24 h	97.88 ± 1.22	97.44 ± 1.75	73.75 ± 9.77	98.41 ± 0.62	97.82 ± 2.30	97.82 ± 1.01	98.59 ± 0.71	98.12 ± 1.73

Frequency of vibrations: I: 2–52 Hz, II: 82–100 Hz; vibrotherapy duration: T1: 10 min, T2: 45 min; body position: A: lying down with lower limbs raised by 20°, B: lying down.

**Table 3:** Wingate test indices measured 24 h after exercise.

Indices	I-T1-A	I-T1-B	I-T2-A	I-T2-B	II-T1-A	II-T1-B	II-T2-A	II-T2-B
TW (kJ)	17.9 ± 3.03	14.74 ± 0.78	14.87 ± 1.03	14.58 ± 0.54	14.39 ± 0.95	14.77 ± 1.28	14.68 ± 1.04	17.70 ± 0.89
PP (W)	880.38 ± 66.79	891.94 ± 31.39	892.13 ± 41.99	879.81 ± 29.35	880.31 ± 46.29	887.06 ± 58.03	887.06 ± 53.07	875.94 ± 65.54
RPP (W·kg^−1^)	11.74 ± 0.59	11.97 ± 0.47	11.79 ± 0.94	11.79 ± 1.01	11.89 ± 0.75	11.68 ± 0.67	11.89 ± 1.14	11.64 ± 0.95
toPP (s)	4.15 ± 0.79	4.05 ± 0.84	3.77 ± 0.51	3.88 ± 0.49	3.88 ± 0.77	3.98 ± 0.63	3.84 ± 0.62	3.78 ± 0.69
tmPP (s)	3.52 ± 1.54	2.60 ± 0.55	2.77 ± 0.28	3.08 ± 0.39	2.83 ± 0.35	2.89 ± 0.69	3.22 ± 0.92	3.45 ± 0.70
IDP (W·kg·s^−1^)	0.299 ± 0.057	0.347 ± 0.059	0.316 ± 0.051	0.332 ± 0.042	0.341 ± 0.029	0.324 ± 0.035	0.351 ± 0.053	0.316 ± 0.048

Frequency of vibrations: I: 2–52 Hz, II: 82–100 Hz; vibrotherapy duration: T1: 10 min, T2: 45 min; body position: A: lying down with lower limbs raised by 20°, B: lying down. TW—total work, PP—peak power, RPP—relative peak power, toPP—time to obtain peak power, tmPP—time to maintain peak power, IDP—power decrease indicator.

**Table 4:** Concentrations of biochemical indices (myoglobin—Mb; creatine kinase—CK; interleukin 1β—Il-1β and interleukin 6—Il-6; hemoglobin—Hb; hematocrit—Hct) measured in sera of participants (n = 16) subjected to vibrotherapy. The results were obtained before (0) and 60 min and 24 h after intensive exercise effort. Variables are shown as mean values ± standard deviation (SD).

		I-T1-A	I-T1-B	I-T2-A	I-T2-B	II-T1-A	II-T1-B	II-T2-A	II-T2-B
Mb ng/mL	0	37.83 ± 4.72	50.12 ± 13.85	49.06 ± 14.43	46.38 ± 14.99	45.87 ± 14.10	42.36 ± 13.94	41.79 ± 12.84	43.00 ± 12.71
	60 min	50.18 ± 16.85	66.83 ± 17.94	66.33 ± 22.50	74.30 ± 20.31	72.55 ± 30.94	66.20 ± 26.58	73.11 ± 32.03	69.53 ± 20.32
	24 h	38.06 ± 7.84	43.92 ± 11.40	47.22 ± 9.62	47.45 ± 13.99	37.13 ± 12.10	54.05 ± 20.98	46.71 ± 15.58	46.86 ± 9.94
CK ng/mL	0	0.65 ± 0.76	0.59 ± 1.01	1.37 ± 1.20	1.08 ± 1.44	1.34 ± 1.19	0.55 ± 0.53	1.60 ± 1.49	1.06 ± 1.00
	60 min	2.04 ± 1.84	2.67 ± 1.71	2.42 ± 1.87	1.29 ± 1.11	1.54 ± 1.90	0.86 ± 0.77	1.94 ± 1.82	1.36 ± 1.66
	24 h	0.97 ± 1.12	1.76 ± 2.29	3.14 ± 2.01	1.62 ± 1.69	1.32 ± 1.39	1.35 ± 1.78	2.69 ± 2.87	1.52 ± 1.48
Il-1β pg/mL	0	1.68 ± 0.44	1.65 ± 0.41	1.79 ± 0.47	1.67 ± 0.62	1.78 ± 0.37	1.79 ± 0.40	1.80 ± 0.62	1.88 ± 0.37
	60 min	1.68 ± 0.45	1.66 ± 0.40	1.81 ± 0.54	1.69 ± 0.58	1.79 ± 0.36	1.85 ± 0.40	1.80 ± 0.64	1.96 ± 0.32
	24 h	1.67 ± 0.42	1.67 ± 0.40	1.82 ± 0.46	1.62 ± 0.65	1.78 ± 0.39	1.79 ± 0.38	1.83 ± 0.62	1.84 ± 0.41
Il-6 pg/mL	0	2.20 ± 0.33	1.90 ± 0.46	2.06 ± 0.18	2.22 ± 0.38	2.21 ± 0.51	2.17 ± 0.29	2.10 ± 0.49	2.18 ± 0.24
	60 min	2.82 ± 0.80	2.15 ± 0.58	2.49 ± 0.25	2.59 ± 0.39	2.70 ± 0.96	2.94 ± 0.93	2.52 ± 0.87	2.45 ± 0.41
	24 h	2.01 ± 0.31	1.75 ± 0.20	2.25 ± 0.29	2.16 ± 0.31	2.13 ± 0.40	2.06 ± 0.34	1.90 ± 0.34	2.14 ± 0.39
Hb g/dL	0	15.95 ± 0.50	15.85 ± 0.85	15.44 ± 0.70	16.15 ± 0.61	15.80 ± 0.69	15.72 ± 0.65	15.61 ± 0.73	15.70 ± 0.64
	60 min	15.84 ± 0.74	15.41 ± 0.94	15.42 ± 0.63	16.24 ± 0.73	15.78 ± 0.73	15.62 ± 0.64	15.45 ± 0.92	15.50 ± 0.72
	24 h	15.90 ± 0.55	15.62 ± 0.88	15.53 ± 0.60	16.12 ± 0.53	15.52 ± 0.62	15.62 ± 0.76	15.36 ± 0.64	15.49 ± 0.74
Hct %	0	45.56 ± 0.89	45.44 ± 1.75	44.62 ± 1.49	46.69 ± 1.78	45.53 ± 1.46	45.23 ± 1.34	44.71 ± 2.19	44.86 ± 1.86
	60 min	45.26 ± 1.20	44.41 ± 1.77	43.91 ± 1.48	46.21 ± 1.60	44.85 ± 1.35	44.40 ± 1.33	44.28 ± 2.10	44.19 ± 1.99
	24 h	45.09 ± 1.20	45.03 ± 1.81	43.78 ± 1.20	45.83 ± 1.50	43.93 ± 1.12	44.86 ± 1.58	43.72 ± 1.60	43.93 ± 1.93

Frequency of vibrations: I: 2–52 Hz, II: 82–100 Hz; vibrotherapy duration: T1: 10 min, T2: 45 min; body position: A: lying down with lower limbs raised by 20°, B: lying down.

## Data Availability

The data presented in this study are available on request from one of the authors (T.P. tomasz.palka@awf.krakow.pl).
